# The Lourdes Pilgrimage and the Impact on Pilgrim Quality of Life

**DOI:** 10.1007/s10943-021-01398-0

**Published:** 2021-09-09

**Authors:** Jennifer Klimiuk, Kieran J. Moriarty

**Affiliations:** 1 Bolton Hospice, Bolton, BL1 4QT UK; 2 Bolton NHS Foundation Trust, Bolton, BL4 0JR UK

**Keywords:** Lourdes, Pilgrimage, Quality of Life

## Abstract

Lourdes, France, is a major site of pilgrimage, particularly for Roman Catholics with illness. The direct impact of pilgrimage on pilgrim quality of life (QOL) has not previously been measured. The present study aimed to measure the impact of pilgrimage to Lourdes on QOL in self-defined “sick pilgrims”. The standardised EuroQol EQ-5D-5L questionnaire measured two aspects of QOL, namely a Visual Analogue Scale (VAS) score of self-rated health and an Index Value Score (IVS) of the five dimensions of QOL, in a group of pilgrims, before (Q1), immediately after (Q2) and two months after (Q3) return from pilgrimage to Lourdes. A total of 93 pilgrims responded at time Q1, 71 at Q2 and 64 at Q3. The VAS scores of self-rated health showed statistically significant improvement at Q2 (*p* = 0.04), although this was not sustained at Q3. The IVS Scores showed no significant differences at Q2 or Q3. However, at Q2, 67.6% of pilgrims reported their self-rated QOL as “much better” or “better”, and this was maintained in 54.7% at Q3. Pilgrims identified “spiritual and religious aspects of pilgrimage”, “a sense of togetherness” and “spiritual healing” as having the most significant impact on their QOL. The Lourdes pilgrimage had a statistically significant positive impact on the immediate post-pilgrimage VAS scores of QOL of “sick pilgrims”, but this was not sustained two months following pilgrimage. The IVS scores were unchanged. Pilgrims identified beneficial holistic, spiritual and communal aspects of the pilgrimage experience.

## Introduction

Lourdes is a small town in the French Pyrenees, with a population of 15,000. The shrine in Lourdes is recognised by the Roman Catholic Church as the site of 18 visions of the Virgin Mary to Bernadette Soubirous in 1858. Since then, Lourdes has been a major site of pilgrimage, especially for Roman Catholics. Lourdes has attracted millions of pilgrims from across the world and is now the largest pilgrimage site in Western Europe. Approximately four to six million pilgrims, many with physical or mental illness, travel to Lourdes each year (Lourdes Sanctuaire, 2019). Whilst pilgrims are aware of reported “cures” in Lourdes, most travel for other reasons, including to experience the transformative atmosphere of Lourdes (Gesler, 1996; Higgins & Hamilton, 2016).

The pilgrimage experience in Lourdes consists of multiple elements. These include prayer and devotion at the Grotto site, where the visions are believed to have taken place and actively participating in daily collective worship, including Holy Mass and processions. Many pilgrims visit the baths and water faucets, which provide water from the spring believed to have been discovered by Saint Bernadette.

Since 1858, only 70 cures at Lourdes have been recognised by the Roman Catholic Church as being medically inexplicable and “miraculous”. However, many thousands of unexplained cures have been reported to the Bureau des Constatations Médicales or Lourdes Medical Bureau (Francois et al., 2012; Lourdes Sanctuaire, 2019).

Anecdotally, many pilgrims benefit from the pilgrimage experience (Lillie, 2005; Morris, 1982), sometimes holistically, although their physical or mental status may remain unchanged. A study by Higgins & Hamilton (2016) suggested the concept of “mini-miracles” to refer to changes that occur in an individual’s life, significant to the individual, but unlikely to be considered “miraculous” by the wider church.

Although previous studies have explored potential benefits of pilgrimage to Lourdes, none has directly measured the impact of pilgrimage to Lourdes on health-related QOL. Since the impact of Lourdes is multifactorial and may include physical, psychological, social and spiritual dimensions, a measure of general health and well-being state, such as QOL, is most likely to accurately capture the effect of these different factors.

## Aims and Objectives of the Present Study

The present study was designed to measure the impact on QOL, and also on the key determinants of QOL, in a group of “sick pilgrims”, before and after travelling on pilgrimage to Lourdes with the diocese of Salford from the North West of England in August 2015.

## Methods

### Study Population

All 416 pilgrims, aged 18 and older, attending the diocese of Salford annual 6-day pilgrimage to Lourdes, were invited to participate in the study. The diocese of Salford includes part of the North West of England, including the city of Manchester.

Pilgrim confidentiality precluded documentation as to how many were “healthy” and how many were “sick pilgrims”. Anyone who defined themselves as a “sick pilgrim”, suffering from chronic ill health, was eligible to participate, although they may not have officially registered with the pilgrimage nursing and medical team as a “sick pilgrim”. This is because they were self-caring, or they may have travelled as a volunteer. For this study, the term “sick pilgrim” was used, as this is a generally accepted international term for any pilgrim, including pilgrims to Lourdes, who suffers from physical and/or mental illness. It is difficult to define what the term “sick” encompasses and there is a consensus that the definition of chronic illness and QOL are the preserve of individuals (Anderson et al., 1991; Funnel et al., 1991; Price, 1996). We did not ask about severity and length of sickness, nor on the intentions of our pilgrims.

### Study Instruments and Statistical Methods

Prior to the pilgrimage, the primary researcher (JK) mailed both a letter of explanation and also the first questionnaire (at Q1) to all 416 pilgrims. It was explained that participants would be asked to complete a series of three identical questionnaires. Informed consent was obtained from all individuals who chose to partake in the study and returned the first questionnaire. Basic demographic data were also collected and pilgrims were asked, if possible, to select the category or categories of their diagnosis or diagnoses.

At Q2, immediately on return from Lourdes, the second questionnaire was mailed to those pilgrims who had consented to participate and completed the first questionnaire. At Q3, two months following return from Lourdes, the third questionnaire was mailed to those pilgrims who had completed and returned the second questionnaire.

On the advice of our university statistician, we used the standardised EuroQol EQ-5D-5L questionnaire (EuroQol Group, 1990; van Hout et al., 2012). This incorporates two measurements of QOL, the Visual Analogue Scale (VAS) score and the Index Value Score (IVS). The VAS score records self-rated health on a vertical visual scale and is a quantitative measure of health outcome, based on the participant’s own judgement.

The second measurement, the Index Value Score (IVS), is a descriptive system containing the five dimensions of QOL, namely mobility, self-care, usual activities, pain/discomfort and anxiety and depression. Each of the five dimensions of QOL is scored from 1 = “no problems” to 5 = “extreme problems”. Participants were asked to record which number was most relevant to their current state of health. The 5 single digit scores for each of the five dimensions (e.g. 2, 3, 5, 1, 4) was the “health profile” for each individual. This was then matched against crosswalk value sets and corresponds to an overall Index Value Score (van Hout et al., 2012).

In the EQ-5D-3L questionnaire, respondents rate their degree of impairment in different health domains using 3 response levels (1 = no problems, 2 = some problems, 3 = extreme problems). The EuroQuol Group created a descriptive system EQ-5D-5L, with 5 response levels (1 = no problems, 2 = slight problems, 3 = moderate problems, 4 = severe problems, 5 = extreme problems). EQ-5D-5L was designed to be more sensitive than EQ-5D-3L. The crosswalk was based on a response mapping approach that estimated the relationship between responses to the EQ-5D-3L and EQ-5D-5L descriptive systems, and subsequently established a link to the 3L value sets (van Hout et al., 2012).

Statistical analysis is included in the EuroQol EQ-5D-5L questionnaire. When the crosswalk value sets were combined with the Student’s paired t-test, this facilitated longitudinal and linear regression data analysis that assessed any changes at the 3 time points, Q1, Q2 and Q3, before, immediately on return and 2 months following return from pilgrimage, respectively. The data were analysed using both parametric and nonparametric approaches. The results were broadly similar, the one statistically significant change using both approaches being in the VAS scores at Q1. The parametric analysis was preferred since the data appeared normally distributed, it provided real data rather than data ranks, and it also facilitated the calculation of standard deviations and confidence intervals.

Whilst it is not part of the EuroQol EQ-5D-5L questionnaire, at Q1, in order to gain an idea of the spectrum of QOL of the participants, we recorded the summated scores for the five dimensions. Therefore, the minimum score could equal 5 and range up to a maximum of 25. Additionally, in questionnaires 2 and 3, participants were asked whether, following the pilgrimage, they felt “much better”, “better”, “the same” or “worse”. At Q2 and Q3, participants were also invited to rank 1, 2 or 3 (1 being the most important) which aspects of pilgrimage they felt had the most impact on their QOL.

## Results

A total of 94 self-defined “sick pilgrims” responded to the questionnaire at Q1. One pilgrim was excluded due to an incomplete form. Of the 93 participants, 57 females and 36 males, 71 (76%) responded at Q2 and 64 of the 71 responded at Q3, two months following return. The age range was 21–94 years, mean age 72. One respondent identified as being of the Church of England faith and 92 were of the Roman Catholic faith.

The group of respondents comprised “sick pilgrims” registered with the nursing and medical team, “hotel” pilgrims travelling independently, pilgrimage volunteers and clergy. The most common diagnostic groups were “heart and vascular problems”, “joint and muscle problems”, “sight or hearing problems”, “endocrine disorders” and “gastrointestinal problems”. Some “sick pilgrims” selected more than one diagnostic category, the 93 participants selecting a total of 153 diagnostic categories, with 14 completing the questionnaire fully, but not selecting a diagnostic category (Table 1). Since the selection of a diagnostic category was our own additional request and not an inherent component of the EuroQol EQ-5D-5L questionnaire, these 14 were included in the analysis.

**Table 1:** Diagnostic groups of participants (Numbers)

Heart & vascular problems (e.g. angina, high blood pressure, heart failure, leg ulcers)	34	Genitourinary problems (e.g. prostate problems, incontinence, endometriosis)	3
Joint & muscle problems (e.g. arthritis, chronic back pain)	29	Kidney problems (e.g. kidney infections, renal failure)	2
Sight or hearing impairment	19	Movement disorder (e.g. Parkinson’s disease, cerebral palsy)	2
Endocrine disorders (e.g. diabetes, thyroid problems)	14	Other forms chronic physical impairment (e.g. paralysis, amputation)	2
Gastrointestinal problems (e.g. indigestion, gastritis, diarrhea, constipation)	13	Cancer	2
Skin problems (e.g. psoriasis, dermatitis, eczema)	9	Other- Chronic Fatigue Syndrome	1
Lung problems (e.g. emphysema, chronic bronchitis, bronchiectasis)	7	Learning disabilities	0
Mood problems or psychiatric problems (e.g. depression, anxiety, schizophrenia, bipolar disorder)	6	Chronic infection (e.g. hepatitis, TB)	0
Neurological problems (e.g. stroke, multiple sclerosis, epilepsy, nerve damage)	5	No category selected	14
Dementia or memory problems	5		

The Visual Analogue Scale (VAS) scores of self-rated health of pilgrims showed a statistically significant improvement in QOL at Q2 (*p* = 0.04). At Q3, whilst there was a trend towards improvement of the VAS scores, this did not achieve statistical significance (*p* = 0.18) (Table 2).

**Table 2:** Visual analogue scores (*n* = 60)

	Q1	Q2	Q3
Minimum score	35	35	35
Maximum score	100	100	100
Mean score	72.8	76.3	75.4
Standard deviation of score	18.0	17.5	17.5
Mean difference	–	3.5	2.7
95% confidence interval for difference	–	0.3–6.7	−1.2–6.5
P-value (paired t-test)	–	0.04	0.18

The Index Value Scores showed no significant differences at Q2 or Q3 (Table 3). The anxiety and depression dimension scores showed a trend towards reduction at Q2, but this was not statistically significant (*p* = 0.11).

**Table 3:** Index value scores (*n* = 54)

	Q1	Q2	Q3
Minimum score	− 0.3	− 0.1	− 0.1
Maximum score	1	1	1
Mean score	0.7	0.7	0.7
Standard deviation of score	0.3	0.2	0.3
Mean difference	–	0.01	− 0.01
95% confidence interval for difference	–	− 0.02–0.05	− 0.04–0.03
*P*-value (paired t-test)	–	0.45	0.78

The summated scores of the five dimensions of QOL ranged between 5 and 19 (mean 8.67, median 8.0), a wide spectrum. At Q2, of the 71 questionnaires returned, 48 (67.6%) reported their self-rated QOL as “much better” or “better”, 20 (28.2%) “the same”, 2 (2.8%) “worse”, with 1 (1.4%) incomplete form. At Q3, from 64 of the 71 questionnaires returned, the respective figures were 35 (54.7%) “much better” or “better”, 23 (35.9%) “the same”, 4 (6.3%) “worse”, with 2 (3.1%) incomplete forms.

At Q2, the three aspects ranked as having the most significant impact on QOL were “spiritual and religious aspects of pilgrimage”, “a sense of togetherness and community” and “a sense of spiritual healing” (Table 4). At Q3, the overall ranking was very similar (Table 5). Moreover, at Q2 and Q3, the three main aspects were identical, accounting for 72% at both Q2 and Q3 of the total number of aspects ranked. At Q2, three pilgrims ranked “Other” aspects, namely “singing in the choir”, “the unique charisma of Lourdes” and “consolidation of family unity”, as impacting positively on their QOL following pilgrimage. At Q3, there was no ranking recorded under “Other” aspects.

**Table 4:** Ranked aspects of the pilgrimage impacting on QOL at time Q2

Reason	RANK 1	RANK 2	RANK 3
Spiritual and religious aspects of pilgrimage	40	6	1
A sense of togetherness/community/being with others	6	17	17
A sense of spiritual healing	3	13	12
Relationships formed during pilgrimage	1	4	7
Holiday	2	3	5
A sense of psychological healing	2	5	2
Social aspects of pilgrimage	0	2	6
A sense of physical healing	0	0	2
Other	0	0	3

**Table 5:** Ranked aspects of the pilgrimage impacting on QOL at time Q3

Reason	RANK 1	RANK 2	RANK 3
Spiritual and religious aspects of pilgrimage	31	10	3
A sense of togetherness/community/being with others	7	15	10
A sense of spiritual healing	4	16	9
Relationships formed during pilgrimage	0	1	14
Holiday	5	1	2
A sense of psychological healing	2	1	2
Social aspects of pilgrimage	0	2	6
A sense of physical healing	2	2	0
Other	0	0	0

## Discussion

Since pilgrim confidentiality precluded documentation as to how many pilgrims were “healthy” and how many were “sick pilgrims”, we were encouraged that 93 “sick pilgrims” responded to the initial invitation and fully completed and returned their questionnaire at Q1.

The present study has shown that pilgrimage to Lourdes had a statistically significant impact on VAS self-rated health scores of “sick pilgrims” at Q2, immediately on their return home from Lourdes. At Q2, 71 of the 93 participants responded, an encouraging number in the context of our pilgrimage. Our findings could reflect that the 22 non-responders did not return their questionnaires because they had failed to improve. Following return home from pilgrimage, some pilgrims may have had other priorities, which could have accounted for some non-responders. We were especially encouraged that 64 of the 71 responders at Q2 again responded at Q3, two months after return, indicating continuing commitment to the study. At Q3, whilst there was a trend towards improvement of the VAS scores, this did not achieve statistical significance (*p* = 0.18). It is possible that a statistically significant change may have been missed due to the dropout rate of around 1/3 of the original sample and the small sample size.

The Index Value Scores showed no significant differences at Q2 or Q3. Since the mean IVS scores were actually identical at Q1, Q2 and Q3, it seems unlikely that the dropout rate and small sample size would have significantly influenced statistical analysis. The summated scores of the five dimensions of QOL showed a wide variation. At Q2, 67.6% reported their self-rated QOL as “much better” or “better”. Whilst this improvement might be expected immediately following a pilgrimage, it was sustained two months later in 54.6%. At Q2 and Q3, the overall rankings of aspects of pilgrimage having the most significant impact on QOL were virtually identical, the three main ones being “spiritual and religious aspects of pilgrimage”, “a sense of togetherness and community” and “a sense of spiritual healing”.

Positive “religious coping”, such as seeking out spiritual support, can have a beneficial impact on health outcomes (Pargament et al., 2004), and prayer and spiritual practice may positively impact on QOL (Jantos & Kiat, 2007). This may be relevant when considering the self-selecting group of pilgrims who travel on pilgrimage to Lourdes. It was not practical to assess the degree of religious commitment or observance of our pilgrims.

“Sick pilgrims” travelling to Lourdes gain a number of positive outcomes from the experience. Morris (1982) found a sustained reduction in the anxiety and depression scores of physically sick patients, both one month and ten months after return from pilgrimage to Lourdes. This was considered to be due to several factors, including the element of a vacation. However, a key finding was that most reported a “strengthened religious faith”, resulting in a greater ability to accept their physical disabilities. The study concentrated on quantifying emotional level in those who were physically sick, and although it suggested that there may be an improvement in quality of life in those who experienced lesser depression or anxiety, this was not directly measured. In our study, anxiety and depression dimension scores of QOL showed a trend towards a reduction at Q2, but this was not statistically significant (*p* = 0.11). Once again, it is possible that a statistically significant change may have been missed due to the dropout rate of around 1/3 of the original sample and the small sample size.

Lourdes is a very social place and pilgrims often spend time in conversation in cafés and bars away from the shrine. In larger pilgrimages, pilgrims may spend their time together, participating in social events, staying in the same hotels and eating meals as a group. In such pilgrimages, volunteer helpers, clergy, medical and nursing staff participate alongside “sick pilgrims”, and normal social barriers are removed. In Lourdes, mass gatherings, collective worship and an atmosphere of shared identity are all an integral part of daily culture. In our study, “a sense of togetherness and community” was a key factor impacting positively on QOL.

Studies of pilgrimages to other religious sites have also highlighted areas of potential perceived benefits. A study of a Hindu pilgrimage by the Prayag Mela Research Group (2007) showed that, despite the difficult pilgrimage conditions, including crowds and sleep deprivation, pilgrims felt a greater sense of well-being after attending. This was largely attributed to a shared-identity and relationality and collective self-realisation. This can be linked to the idea of “social-cure”. Tewari et al. (2012) explored the idea that mass gatherings may have an impact on well-being and found a longitudinal increase in well-being relative to those who did not attend a North Indian Hindu pilgrimage event.

Goldingay et al. (2014) explored the experiences of three academic practitioners in Lourdes. All agreed that aspects of their experiences were complex and inexplicable. One pilgrim reported to them “I felt just complete calm and peace and the pain just disappeared into insignificance”. They concluded that the phenomenon of a collective, shared and embodied purpose was integral to the healing power of Lourdes on the cared for and the carers alike.

In 2014, Francois et al. reviewed a series of physical cures that were reported to the Lourdes Medical Bureau between 1909 and 1976. They stated that *“exposure to Lourdes and its representations …. induced exceptional, usually instantaneous symptomatic, and at best physical, cures of widely different diseases”.* They recognised the differing points of view on this issue, namely those who believed that such cures are “miraculous” and a product of divine intervention, and those who believe that a scientific explanation is yet to be found, possibly a product of complex “somatic and mental processes”, created by the atmosphere in Lourdes. Either way, they agreed that Lourdes was a place of considerable scientific interest and that understanding these healing processes could bring about new and effective therapeutic methods, using the phrase “*The Lourdes Cures concern Science as well as religion”.*

Lillie (2005) examined why palliative care healthcare professionals felt that pilgrimage to Lourdes could be a beneficial activity for the terminally ill. In the present study, we did not ask pilgrims to record if they had a terminal illness. The perceived benefits, which were also seen in our study, included an improvement in quality of life through maintaining patient choice and autonomy, enabling inner-transformations and communitas, altered relationships formed during pilgrimage and the experience of a vacation, albeit often a very tiring one.

In a seminal paper, Sloan et al. (2000), a group of biomedical researchers and chaplains in health care settings, representing a wide range of religious traditions, reviewed the evidence-base for the question “Should Physicians Prescribe Religious Activities?” The authors considered the link between religion and health, whether doctors should recommend religious activity to provide comfort, whether patients wanted religious matters to be discussed and incorporated into their medical care, and the potential issue of “trivialising religion”. The authors challenged assumptions and limitations in previous studies and emphasised the need for collaborative, multidisciplinary research in this controversial area.

Virtual pilgrimages to Lourdes have always been popular, especially in the USA, given the long distances to travel to Lourdes, particularly for “sick pilgrims”. In the Covid-19 pandemic, live streaming of religious activities and virtual pilgrimages to Lourdes and other holy places have become the “new normal”. This will continue for the foreseeable future and for many, especially older, vulnerable “sick pilgrims”, will be their future religious devotion of choice.

Mróz (2021), in one of the first published studies of the impact of the Covid-19 pandemic, showed that pilgrimages and religious tourism to selected European Catholic pilgrimage sites, including Lourdes, decreased by 90–95% during March-September 2020, the first six months of the pandemic. In the two months between mid-March and May, 95% of pilgrimages to Lourdes were cancelled. Shrines of many religions encouraged pilgrims to deepen their bonds with shrines through participation in online services and prayers and spiritual and virtual pilgrimages. Pilgrims who participated spiritually in Lourdes could watch devotions at the grotto site, Holy Mass, blessing of the sick and the evening torchlight procession with candles. In July 2020, the first international virtual pilgrimage in the history of the sanctuary was organised in Lourdes. One potential positive aspect of the pandemic for all religions would be a sustained increase in individual pilgrimages and visits to regional, local and national pilgrimage centres. This may encourage people to become closer and care for those who are ill; support shrines financially and begin to plan future pilgrimages in safety. This study should stimulate further research on the future impact of the Covid-19 pandemic on pilgrimages and religious events, especially following global vaccination programmes.

## Limitations

Limitations to our study need to be considered. One limitation, and of similar studies, is that pilgrims are usually self-selected and pre-primed by their religious beliefs, which can influence perceptions of the factors influencing Quality of Life. The convenience nature of the sample recruitment of pilgrims may have been a factor affecting the generalizability of the study results. The dropout rate of nearly 1/3 from the original sample size may have limited the typical power to detect statistically significant differences in QOL. Moreover, not all participants registered with the nursing and medical team, even though all self-defined as “sick pilgrims”. Whilst there could have been significant differences in the seriousness of illnesses and disabilities that would have affected the statistical results, our study precluded medical assessment of disease severity, prior to or following pilgrimage.

The EuroQol EQ-5D-5L questionnaire was chosen, since it is validated and does cover 5 dimensions of QOL. Moreover, it was felt to be the most appropriate for our pilgrim cohort. The dropouts at Q2 and Q3 could have been the disappointed pilgrims, who had not improved, which might lead to an interpretation bias. Whilst it would have been interesting to examine the impact of pilgrimage on the different diagnostic groups, this was not performed as many pilgrims selected more than one medical condition. In retrospect, statistical analysis using the Friedman test may have facilitated data interpretation (Friedman, 1937, 1939).

## Conclusion

Our study adds to the evidence-base and literature, in that the VAS scores of Quality of Life of “sick pilgrims” showed statistically significant quantitative improvement immediately after our Lourdes pilgrimage, although this was not sustained and the Index Value Scores showed no significant differences. Short-term improvements after travel to sacred places are usually a matter of being emotionally “moved” by the experiences in a larger community, and these tend to decrease once the pilgrim is back home in “ordinary” life, where they once again tend to focus on being “sick”, in isolation and without the support of fellow pilgrims or other people.

The three factors identified as having the most significant impact on QOL were “spiritual and religious aspects of pilgrimage”, “a sense of togetherness” and “spiritual healing”, highlighting the holistic, spiritual and communal aspects of the pilgrimage experience. Our findings also support the need for further research to explore this complex subject, which extends across physical, psychological, social, religious and spiritual dimensions. Moreover, future research will need to include study of the physical, mental, emotional, spiritual and holistic benefits and disadvantages of both physical and virtual pilgrimages and live streamed services for people and pilgrims of a wide range of ages and vulnerabilities.
